# Prevalence of Serotonergic Drug Use in Patients Exposed to Perioperative Methylene Blue: A Cross-Sectional Study

**DOI:** 10.7759/cureus.81090

**Published:** 2025-03-24

**Authors:** Elijah McMillan, Shawn Meepagala, Kamdili Ogbutor, Da'Jhai Monroe, Trinity Gibbs, Noah Wheaton, Nurupa Ramkissoon Ramkissoon, Samrawit W Zinabu, Miriam B Michael

**Affiliations:** 1 Anesthesiology and Critical Care, Howard University College of Medicine, Washington, DC, USA; 2 Pathology, Howard University Hospital, Washington, DC, USA; 3 Internal Medicine, Howard University Hospital, Washington, DC, USA; 4 Anesthesia, Howard University Hospital, Washington, DC, USA; 5 Obstetrics/Gynecology, Howard University Hospital, Washington, DC, USA; 6 Internal Medicine, Howard University, Washington, DC, USA; 7 Internal Medicine, University of Maryland, Baltimore, USA

**Keywords:** methemoglobinemia, methylene blue, serotonergic drug, serotonergic drug interaction, serotonergic medications

## Abstract

Background: Methylene blue is a widely used medication in various medical procedures, particularly in cardiovascular operations where it serves as a diagnostic dye and a treatment for conditions like methemoglobinemia and vasoplegic syndrome. While its clinical applications are well-documented, methylene blue's pharmacological properties as a monoamine oxidase (MAO) inhibitor present a potential risk of serotonin syndrome when administered to patients taking serotonergic medications. This study examines the prevalence of serotonergic medications, including lithium, selective serotonin reuptake inhibitors (SSRIs), serotonin-norepinephrine reuptake inhibitors (SNRIs), tricyclic antidepressants (TCAs), and monoamine oxidase inhibitors (MAOIs), in patients who were administered methylene blue.

Methods: This cross-sectional study used de-identified electronic health records (EHRs) from the TriNetX database, which pools data from 97 healthcare organizations. The analysis focused on isolating patients who were administered methylene blue perioperatively and calculating the prevalence of concomitant serotonergic drug use by drug class.

Results: The large database analysis revealed that among the 249,131 patients who received perioperative methylene blue, 25,282 (10.14%) were concomitantly given serotonergic drugs. By drug class, SSRIs had the highest concomitant use with 15,705 cases and a prevalence of 63.04/1000 cases. Next were SNRIs, with 6,341 cases and a prevalence of 25.45/1000 cases; TCAs, with 2,979 cases and a prevalence of 11.96/1000 cases; lithium, with 292 cases and a prevalence of 1.17/1000 cases; and MAO inhibitors, which had the lowest concomitant use at 65 cases and a prevalence of 0.26/1000 cases.

Conclusion: The prevalence of serotonergic agent use in patients administered methylene blue is relatively high exceeding 10%, with the highest prevalence observed in those on SSRIs, followed by SNRIs and TCAs. This underscores the importance of a thorough preoperative evaluation by an anesthesiologist to minimize the risk of intraoperative complications such as serotonin syndrome.

## Introduction

Methylene blue is an FDA-approved drug that is used for the treatment of methemoglobinemia. However, it is also commonly used for off-label use as a dye in surgeries, particularly in procedures like parathyroid surgery, where it helps to identify abnormal tissue [1]. Its utility extends to other surgeries such as thyroid, colorectal, breast, renal, and cardiac procedures [1-3]. However, while methylene blue’s clinical benefits are well-established, its pharmacological properties as a monoamine oxidase (MAO) inhibitor introduce risks when administered to patients taking serotonergic medications. As the prevalence of serotonergic medications (e.g., lithium, selective serotonin reuptake inhibitors (SSRIs), serotonin-norepinephrine reuptake inhibitors (SNRIs), tricyclic antidepressants (TCAs), and monoamine oxidase inhibitors (MAOIs)) continues to rise, understanding the associated risk of serotonin syndrome during surgical procedures involving methylene blue is essential to ensuring patient safety.

Serotonin syndrome is a potentially life-threatening condition resulting from excessive serotonin accumulation in the central nervous system. It is a rare condition; however, it has been shown to occur in around 12% of patients in the inpatient psychiatric setting and around 7.8% of patients in the intensive care unit (ICU) [4,5]. It is often underdiagnosed due to variability with diagnostic criteria and overlapping symptoms with other conditions [6]. Symptoms can range from mild to severe in intensity and include hyperthermia, confusion, muscle rigidity, and autonomic instability. This syndrome often arises from the combination of multiple serotonergic agents. It has been seen that serotonin syndrome can result when methylene blue, which inhibits serotonin breakdown through its MAO inhibitor activity, is given to patients who concurrently take medications with serotonergic activity [3,7].

A dose as small as 0.75 mg/kg of methylene blue has been shown to reach a high enough concentration within the central nervous system that would inhibit monoamine oxidase and put a patient at an increased risk for serotonin syndrome [8]. Analysis has shown that there has been an increase in the number of prescriptions of serotonergic agents in the United States from 1996 to 2015 with increases in prevalence of 6.1% to 10.4%, respectively [9]. Given that serotonergic drugs are frequently prescribed in modern clinical practice, understanding the interactive risk of serotonin syndrome when using methylene blue is critical.

This cross-sectional study aims to assess the prevalence of serotonergic drug use among perioperative methylene blue recipients. The serotonergic drugs that were assessed in this study were lithium, SSRIs, SNRIs, TCAs, and MAOIs. By determining the prevalence of these occurrences, we aim to emphasize the importance of a comprehensive preoperative review of a patient’s medication list by both the anesthesiologist and surgeon to mitigate the potential for serotonin toxicity.

We hypothesize that there is a notable prevalence of perioperative methylene blue administration in patients simultaneously using serotonergic agents, leading to a higher risk of serotonin syndrome.

## Materials and methods

In this cross-sectional study, we queried a clinical database containing records of 120,000,000 patients with documented encounters globally from January 1, 2004, to December 31, 2023. Patient data were obtained using the TriNetX database, which aggregates records from 97 healthcare organizations (HCOs). The study used de-identified data and involved no human-subject interactions; therefore, it is exempt from IRB review per Health Insurance Portability and Accountability Act (HIPAA) Privacy Rule standards.

To be included in this study, patients must have received perioperative methylene blue while also being on a serotonergic agent during a surgical procedure. Perioperative methylene blue administration was defined as sole intraoperative ​​​administration of methylene blue through intravenous or oral routes. Cases in which methylene blue was not administered on the same day as their procedure, preoperatively, or postoperatively were excluded from this study.

Data analysis consisted of two main steps, which included defining the cohorts and setting up and running the analysis. To define the cohorts, patients were included based on query criteria. Query parameters consisted of methylene blue (NLM: RXNORM code: 6878) and surgery (UMLS:CPT: 1003143). Setting up and running the analysis consisted of including the index event (first occurrence of the selected cohort criteria), time window (outcomes were analyzed from the same day as the index event), and the outcome criteria.

The outcome criteria consisted of quantifying the prevalence of concurrent serotonergic use among perioperative methylene blue recipients. Patients were grouped by drug class (SSRIs, SNRIs, TCAs, lithium, and MAOIs) and analyzed for prevalence rates.

Descriptive statistics were used to determine the prevalence of concomitant serotonergic agent use, expressed as cases per 1,000 patients. Prevalence rates were calculated for each drug class (SSRIs, SNRIs, TCAs, lithium, and MAOIs). Comparative prevalence rates were presented, and gender- and race-based prevalence rates were analyzed using subgroup comparisons. All statistical analyses were conducted using the TriNetX platform.

## Results

This analysis found 249,131 patients (Tables 1, 2) who received methylene blue during surgery between January 1, 2004, and December 31, 2023. Among these patients, serotonergic agents were concurrently used in 25,282 patients given perioperative methylene blue, indicating a prevalence of 10.14%. The prevalence of concomitant use of a serotonergic agent and perioperative methylene blue was then measured by drug class. SSRIs had the highest concomitant use at 15,705 cases with a prevalence of 63.04/1000 cases. SNRIs had the next highest concomitant use at 6,341 cases with a prevalence of 25.45/1000 cases; TCAs had 2,979 cases with a prevalence of 11.96/1000 cases; lithium had 292 cases with a prevalence of 1.17/1000 cases; and finally MAOIs, with 65 cases, had the lowest prevalence of 0.26/1000 cases.

**Table 1:** Study demographics

Total cases of methylene blue administration	Total cases of methylene blue administration with simultaneous serotonergic agent intake	Percentage of methylene blue with simultaneous serotonergic agent intake (%)
249,131	25,282	10.14

**Table 2:** Occurrences of perioperative methylene blue administration with simultaneous serotonergic agents by subgroup SSRI: Selective serotonin reuptake inhibitor; SNRI: Selective norepinephrine reuptake inhibitor; TCA: Tricyclic antidepressant; MAOI: Monoamine oxidase inhibitor.

Cohort	Patients	Percentage of population (%)	Prevalence rate by 1000
Lithium	292	0.12	1.17
SSRI	15,705	6.30	63.04
SNRI	6,341	2.54	25.45
TCA	2,979	1.20	11.96
MAOI	65	0.03	0.26

Regarding patient demographics (Table 3), females had a higher average prevalence rate (per 1,000) of concomitant serotonergic agent use while being administered perioperative methylene blue at 0.10% compared to male subjects at 0.03%. Furthermore, white subjects and American Indian or Alaska Native subjects had the highest prevalence rates with 0.10% and 0.11%, respectively.

**Table 3:** Prevalence rate of serotonergic drug usage for patients who were administered perioperative methylene blue by race and gender SSRI: Selective serotonin reuptake inhibitor; SNRI: Selective norepinephrine reuptake inhibitor; TCA: Tricyclic antidepressant; MAOI: Monoamine oxidase inhibitor.

Cohort	Females (per 1,000)	Males (per 1,000)	Black or African American (per 1,000)	White (per 1,000)	Asian (per 1,000)	American Indian or Alaska Native (per 1,000)
Lithium	.008	.005	.005	.007	.001	.012
SSRI	.273	.016	.187	.257	.098	.256
SNRI	.147	.076	.103	.131	.046	.148
TCA	.098	.058	.087	.084	.040	.109
MAOI	6.15E-5	1.15E-4	0	5.54E-5	0	0

In summary, the use of serotonergic agents in patients receiving methylene blue varies across these serotonergic drug groups, with SSRIs having the highest concurrent use. For every 1000 patients who had surgery with methylene blue, around 63 were concurrently using SSRIs. This analysis highlights the importance of preoperative testing and careful monitoring of patients on serotonergic medications who receive methylene blue to mitigate the risk of serotonin syndrome.

## Discussion

Summary of main findings

Data analysis from the TriNetX database revealed that 10.14% of the 249,131 patients administered methylene blue during surgery from January 2004 to December 2023 were concurrently taking a serotonergic drug. While the literature does not clearly define the incidence of methylene blue-induced serotonin syndrome, the greater than 10% use of serotonergic drugs in patients receiving methylene blue is concerning.

Comparison with other work

While the literature is not extensive in this field, studies have reported neurological events and serotonin syndrome in patients who were given methylene blue while taking serotonergic medications. In a study conducted by Kartha et al., out of a cohort of 193 patients undergoing parathyroidectomy with methylene blue localization, 12 patients were identified to have postoperative neurological events [2]. All 12 patients who experienced neurological events were simultaneously taking an SSRI. In a systematic review conducted by Patel et al., 25 patients were identified as having postoperative neurotoxicity, and all of these patients were found to have been concurrently taking serotonergic medication [10]. A case report and literature review by Schumacher et al. showed 14 cases where patients who take an SSRI or SNRI experienced serotonin syndrome after receiving methylene blue for cardiovascular procedures [3]. While our findings do not report the incidence of serotonin syndrome, they coincide with these studies' findings in further highlighting the occurrence of concurrent serotonergic drug usage in patients receiving methylene blue.

Limitations of the study

This study has some limitations. The data is aggregated from administrative data collected from 93 healthcare organizations (HCOs), and differences in data collection and reporting create a potential for inconsistencies in reporting. Additionally, while this study documents the rate of concurrent usage of medications that cause serotonin syndrome, the study does not track the incidence of serotonin syndrome itself among this population. Patient diagnoses are listed in the form of ICD-10 (International Classification of Diseases 10th Revision) codes, and the ICD-10 code for serotonin syndrome G90.81 was made effective on October 1, 2024, thus limiting the data on serotonin syndrome incidence [11]. Also, the dosing of methylene may have varied, as the amount of methylene blue administered was not reported in the TriNetX database. Another limitation is the reliance on electronic health records (EHRs) and ICD coding, which may introduce reporting bias or incomplete medication histories. Further study is necessary to evaluate if concomitant use of intraoperative methylene blue in patients taking serotonergic agents has led to an increased incidence of serotonin syndrome. Due to these limitations, this study cannot prove causality between the aforementioned serotonergic agents and the occurrence of serotonin syndrome.

Clinical implications

A study by Treviño et al. demonstrated that benzodiazepines and SSRIs are among the most frequently prescribed medications for Major Depressive Disorder (MDD), accounting for over 40% of the study population [12]. Current literature reports a prevalence of approximately 10.4% for MDD in the United States [13]. Given the widespread diagnosis of MDD and the preference for SSRIs as a first-line treatment, these patients are inherently at higher risk for serotonin syndrome when perioperative administration of methylene blue is anticipated. Despite the FDA requiring a black-box warning on all methylene blue due to its effects when given with another serotonergic agent, there is an alarming occurrence of simultaneous use.

The concomitant use of serotonergic drugs alongside methylene blue also requires the anesthesiologist to have a thorough understanding of the requirements of the surgical case, specifically if methylene blue will be administered. Methylene blue is widely used in various surgical specialties, including ENT, OB-GYN, pulmonary, renal, and cardiac procedures. Specific uses include parathyroidectomy, laparoscopic chromopertubation, pulmonary segmentectomy, and managing vasoplegic syndrome in cardiac surgery [14-16]. It is important to note that methylene blue is not an inert substance. While effective, methylene blue is highly active and poses risks, such as phototoxicity hyperpyrexia, which has led to tissue damage in some parathyroidectomy cases [16,17]. Its role in vasoplegia management involves increasing vascular resistance by inhibiting nitric oxide synthase and guanylate cyclase [18,19].

The responsibility of preoperative evaluation typically falls on the anesthesiologists and surgeons, who must identify any potential risks and perform appropriate risk stratification. If any risks are identified for elective surgery, recommendations should be made to discontinue serotonergic medication two weeks before surgery due to a slow clearance. Although some surgical interventions are time-sensitive and preclude extensive evaluation, when surgery is not urgent, anesthesiologists and surgeons should meticulously review the patient's medication history. If unable to discontinue serotonergic agents before administration of methylene blue, it is recommended to use the lowest possible dosage to mitigate the risk of adverse events. This allows for a thorough risk assessment to reduce the likelihood of intraoperative serotonin syndrome when methylene blue is administered.

## Conclusions

Despite methylene blue carrying a "black-box warning" against use with serotonergic agents, our analysis found a relatively high prevalence of their concomitant perioperative use. The highest prevalence of concomitant use was observed in patients on SSRIs, followed by SNRIs and TCAs. These prevalences underscore the importance of a thorough preoperative evaluation by an anesthesiologist to minimize the risk of intraoperative complications. Future prospective studies or dose-response analyses are warranted to further these findings and investigate the casualty of serotonergic agents and adverse events when paired with methylene blue.
